# Making data map-worthy—enhancing routine malaria data to support surveillance and mapping of *Plasmodium falciparum* anti-malarial resistance in a pre-elimination sub-Saharan African setting: a molecular and spatiotemporal epidemiology study

**DOI:** 10.1186/s12936-022-04224-4

**Published:** 2022-06-29

**Authors:** Frank M. Kagoro, Elizabeth Allen, Aaron Mabuza, Lesley Workman, Ray Magagula, Gerdalize Kok, Craig Davies, Gillian Malatje, Philippe J. Guérin, Mehul Dhorda, Richard J. Maude, Jaishree Raman, Karen I. Barnes

**Affiliations:** 1 Collaborating Centre for Optimising Antimalarial Therapy (CCOAT), Division of Clinical Pharmacology, Department of Medicine, University of Cape Town (UCT), Cape Town, South Africa; 2 Mahidol Oxford Tropical Medicine Research Unit (MORU), Faculty of Tropical Medicine, Mahidol University, Bangkok, Thailand; 3 WorldWide Antimalarial Resistance Network (WWARN), Southern African Regional Hub, Division of Clinical Pharmacology, Department of Medicine, UCT, Mbombela, South Africa; 4 Infectious Diseases Data Observatory (IDDO), Nuffield Department of Medicine, University of Oxford, Oxford, UK; 5 Centre for Tropical Medicine and Global Health, Nuffield Department of Medicine, University of Oxford, Oxford, UK; 6 Mpumalanga Provincial Malaria Elimination Programme, Mbombela Mpumalanga, South Africa; 7 Malaria Programme, Clinton Health Access Initiative, Pretoria, South Africa; 8 Harvard TH Chan School of Public Health, Harvard University, Boston, MA USA; 9 The Open University, Milton Keynes, UK; 10 Centre for Emerging Zoonotic and Parasitic Diseases, National Institute for Communicable Disease, Johannesburg, Gauteng South Africa; 11 Wits Research Institute for Malaria, Faculty of Health Sciences, University of Witwatersrand, Johannesburg, South Africa; 12 UP Institute for Sustainable Malaria Control, Faculty of Health Sciences, University of Pretoria, Pretoria, South Africa

**Keywords:** Artemisinin resistance, *Kelch 13, k13*, Lumefantrine, *Plasmodium falciparum*, Africa, Spatiotemporal model, Malaria

## Abstract

**Background:**

Independent emergence and spread of artemisinin-resistant *Plasmodium falciparum *malaria have recently been confirmed in Africa, with molecular markers associated with artemisinin resistance increasingly detected. Surveillance to promptly detect and effectively respond to anti-malarial resistance is generally suboptimal in Africa, especially in low transmission settings where therapeutic efficacy studies are often not feasible due to recruitment challenges. However, these communities may be at higher risk of anti-malarial resistance.

**Methods:**

From March 2018 to February 2020, a sequential mixed-methods study was conducted to evaluate the feasibility of the near-real-time linkage of individual patient anti-malarial resistance profiles with their case notifications and treatment response reports, and map these to fine scales in Nkomazi sub-district, Mpumalanga, a pre-elimination area in South Africa.

**Results:**

*Plasmodium falciparum* molecular marker resistance profiles were linked to 55.1% (2636/4787) of notified malaria cases, 85% (2240/2636) of which were mapped to healthcare facility, ward and locality levels. Over time, linkage of individual malaria case demographic and molecular data increased to 75.1%. No artemisinin resistant validated/associated * Kelch-13* mutations were detected in the 2385 PCR positive samples. Almost all 2812 samples assessed for lumefantrine susceptibility carried the wildtype *mdr*86ASN and *crt*76LYS alleles, potentially associated with decreased lumefantrine susceptibility.

**Conclusion:**

Routine near-real-time mapping of molecular markers associated with anti-malarial drug resistance on a fine spatial scale provides a rapid and efficient early warning system for emerging resistance. The lessons learnt here could inform scale-up to provincial, national and regional malaria elimination programmes, and may be relevant for other antimicrobial resistance surveillance.

**Supplementary Information:**

The online version contains supplementary material available at 10.1186/s12936-022-04224-4.

## Background

Malaria has been declining globally, with a 50% reduction in malaria cases and an 84% reduction in malaria deaths from 2000 to 2015 [1]. Unfortunately, there has been no significant progress in reducing the global malaria burden since 2015 [2]. The emergence of SARS-CoV-2 threatens to reverse any such progress. The World Health Organization (WHO) estimates an additional 47,000 deaths in 2020 linked to pandemic-related disruptions, with the WHO African region accounting for the majority of these additional cases [3]. The emergence and spread of anti-malarial drug resistance threaten malaria control and elimination efforts, especially in Southeast Asia (SEA), where parasites resistant to artemisinin-based combination therapy (ACT) have been confirmed in at least five countries [4, 5], with resistance markers also reported in China-Myanmar border [6] and eastern India [7]. The majority of SEA countries have low to very low malaria transmission intensities (and thus populations are non-immune), with infections occurring primarily in highly mobile populations along international borders. Several malaria-endemic southern African countries now have similar epidemiological profiles, placing them at increased risk for the emergence of artemisinin (and partner drug) resistance [8, 9]. In sub-Saharan Africa, mutations in the *Plasmodium falciparum Kelch-13* gene (*k13*) associated with artemisinin resistance have been identified in Central (Democratic Republic of Congo) [10], Eastern (Kenya, Rwanda and Tanzania) [11–13] and Western Africa (Ghana, Mali and Nigeria) [13, 14], with phenotypic evidence of artemisinin resistance (delayed parasite clearance) recently confirmed in Rwanda [15] and Uganda [16]. Moreover, Carbo Verde, Eritrea and Ghana were identified as having more than 5% *k13* mutations. More importantly, the *k13* 561HIS mutation in Rwanda [15] and the 469TYR and 675VAL mutations in Uganda have been documented in up to 20% of infected individuals [17]. These three mutations have been associated with reduced efficacy to artemisinin both in-vitro and in-vivo [12, 16, 18]. Recent studies have demonstrated the independent emergence of artemisinin resistance molecular markers in Guyana [19], and the presence of novel *k13* mutations in Brazil [20] and Colombia [21]. In their 2021 systematic review of *k13* markers frequencies in Africa, Ndwiga et al. highlighted the fact that while many African countries were able to identify the *k13* resistance markers using genomic analyses [22], this genomic surveillance was rarely linked to a public health surveillance system.

Robust drug resistance monitoring is a significant challenge, especially in low to moderate malaria transmission settings. While therapeutic efficacy studies (TES) are more feasible in moderate-to-high transmission areas where the required sample sizes can readily be achieved, low and very low transmission settings face recruitment challenges due to fewer malaria cases leading to prolonged study duration, multiple study sites and increased study costs. In such settings, the WHO recommends integrated drug efficacy surveillance (iDES), integrating surveillance of anti-malarial drug efficacy within malaria case-based surveillance [23]. However, resource constraints limit follow up of all malaria cases. This is seldom feasible for mobile and migrant populations, so many low transmission countries fail to monitor anti-malarial efficacy adequately. As of April, 2022, only China [24] and Thailand [25] had published iDES results since its recommendation in 2018 [26]. As countries move towards malaria elimination, many of those with low to very low case numbers need alternative anti-malarial drug resistance surveillance methods. Surveillance of molecular markers associated with drug resistance collected in different clinical trials and observational research has proved useful. However, these studies are generally short-term and are conducted in a few sites where they are not repeated regularly enough to track resistance trends over longer time periods [27]. Integrating sample collection for monitoring molecular resistance markers into routine malaria case surveillance by national malaria programmes has been suggested as a suitable alternative to provide early warning of emerging resistance [28–30]. Such molecular marker surveillance using routine data could then trigger and target transmission-blocking interventions, such as single low dose (SLD) primaquine and foci clearing, and confirmatory therapeutic efficacy studies needed to inform the effective anti-malarial treatment policies essential for achieving elimination. In a case of imported malaria, the malaria programme at the source of the infection can also be informed to trigger and target similar interventions in the source community.

Health information systems used in malaria, such as “District Health Information System 2” (DHIS2), have functionalities that display maps at the ward, district, province, or national malaria programme levels. Whether thematic or modelled, the accuracy of a map depends on the accuracy and reliability of the source data [31–33]. While the need for maps showing the distribution of parasites (malaria cases), vectors and vector breeding sites and prevalence of insecticide and anti-malarial resistance was realized more than two decades ago, data verification, quality assessment and consideration of malaria programme needs to make these malaria maps user-friendly have rarely been included [34]. ‘Human-centred design’ is a part of design thinking that incorporates users in the design process [35, 36]. The co-designing pathway allows a flow of knowledge to both designers and users from development to deployment [36]. Analysing trends in routine data and reviewing results together with the end-users can help improve the data by informing the co-development of tools and resources needed to appraise and enhance data quality, and "Make Data Map-worthy".

In South Africa, all suspected malaria cases should have a definitive diagnosis confirmed by malaria rapid diagnostic test (RDT) or microscopy before treatment is administered [37]. In pre-elimination settings, the Malaria Elimination Programme (MEP) implements both proactive case detection (screening populations at highest risk, such as migrant and mobile populations), as well as reactive case detection (in households surrounding of an index cases’ residence), in addition to passive case detection of patients presenting to health care facilities [38]. The Mpumalanga MEP has stratified Nkomazi sub-district as being in the pre-elimination phase, and has piloted two interventions to enhance monitoring of anti-malarial efficacy and advance malaria elimination. The first was Smart Surveillance for Malaria Elimination (SS4ME), which started in February 2018, and comprised the collection of RDTs (and, wherever possible capillary blood filter paper samples) for tracking molecular markers of anti-malarial resistance. The second was the programmatic roll-out of SLD primaquine recommended by the WHO for malaria transmission blocking [38], in addition to routine treatment of uncomplicated malaria with the artemisinin-based combination therapy, artemether-lumefantrine (AL) [37]; this included WHO recommended follow up of treatment adherence and response during malaria case investigations (from January 2019).

The present study aimed: 1) to map linked patient demographic, clinical and drug resistance profiles in order to identify areas where additional surveillance or containment efforts are needed; 2) to evaluate and quantify the feasibility of this approach, which led to the development of data improvement tools and activities to better meet the needs of the MEP; and 3) to optimize spatial and temporal maps for use by policymakers in local, provincial and national malaria programmes in South(ern) Africa.

## Methods

### Design

A sequential explanatory mixed-methods approach, with iterative quantitative and qualitative methods, was used from March 2018 to February 2020 to optimize maps that linked patient demographic, clinical and drug resistance profiles in order to identify areas where additional surveillance and/or containment efforts are needed. The quantitative component was grounded in the post-positivist theory, where a descriptive and exploratory spatiotemporal analysis was conducted, using trend and time-series decomposition analyses [39] to define spatial and temporal patterns for data linkage and mapping [40]. The qualitative component used a pragmatist approach with co-design techniques to innovate and implement tools to bridge gaps identified from the ongoing spatiotemporal activities and analysis. The MEP and study team worked together iteratively to improve the information system, data architecture and maps produced.

**Fig. 1:**
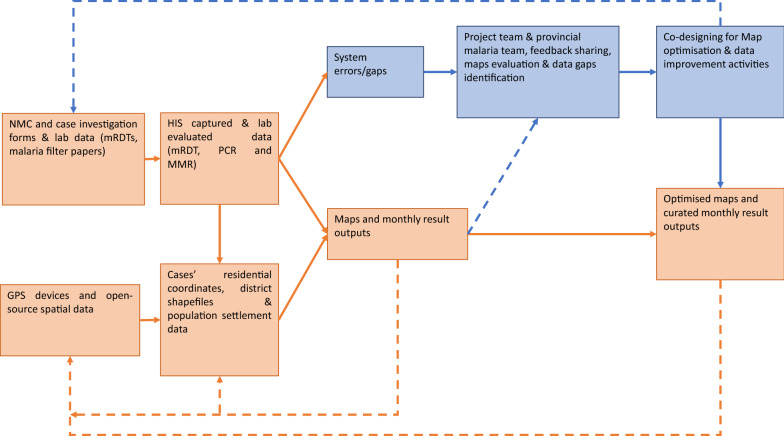
Making data map-worthy study design. Chart showing different iterations of data curation and map optimization. Orange and blue colours show quantitative and qualitative methods, respectively. Solid lines indicate analysis and optimization pathways, while dashed lines show the iteration pathway. *NMC* notifiable medical condition, *HIS* health information system, *RDT* malaria rapid diagnostic test, *PCR* polymerase chain reaction, *MMR* molecular markers of resistance

The quantitative component included data aggregations, curation, and analyses to generate data visualizations. These visualizations and summaries of the analyses were shared monthly with the MEP. The study team then worked with the MEP to design activities, tools and training to enhance data quality and improve surveillance metrics, including coverage, accuracy and linkage (Fig. 1). Data were grouped monthly and quarterly to estimate trends. Monthly evaluations focused on measuring changes in data from the health information system over time. Quarterly evaluations were used to compare the flow of data and accuracy of GPS coordinate data over time.

Spatiotemporal information in the malaria routine case data was used to produce draft maps of malaria incidence and prevalence of molecular markers of resistance. These maps were then presented to the provincial malaria team to evaluate their ‘understandability’, using semi-structured interviews and feedback meetings. These understandability assessments fed back iteratively into the analysis and co-design process until the final maps were agreed upon between researchers and the MEP. This optimization process involved repeatedly deploying and updating the tools and maps produced to enhance routine data and optimize the maps generated.

### Study setting

While most of South Africa is considered malaria-free, approximately 5 million South Africans (10% of the country’s population) reside in the malaria-endemic areas of Mpumalanga, Limpopo, and KwaZulu-Natal provinces [41]. Most malaria cases in South Africa are imported, with some local transmission occurring in the low-altitude [42] international border regions shared with Botswana, Eswatini, Mozambique and Zimbabwe. Malaria transmission in South Africa is seasonal, occurring mainly during the summer rainy season (September to April) [43].

This study was conducted in Nkomazi Sub-District, a pre-elimination area in Mpumalanga province, South Africa. All individuals identified using either proactive, active or passive case detection were tested for malaria using a falciparum-specific, histidine-rich protein 2 (HRP2)-based RDT, and positive cases were included whether or not they were symptomatic [37]. As per the national treatment guidelines, those with asymptomatic or uncomplicated malaria are treated with the WHO recommended weight-based 3day AL (Coartem^®^) regimen [37]. AL has been used in the study area since 2007 [27, 37, 42]. Additionally, all consenting malaria-positive patients, excluding pregnant women, breastfeeding mothers and children under 10 kg or one year of age, are given a single low dose of primaquine (0.25 mg base/kg /15 mg base adult maximal dose) [44–46]. An additional dried blood spot (DBS) on filter paper (Whatman Paper No 1) was collected from RDT malaria positive patients by dabbing the remaining blood at the RDT finger prick site, then labelled, barcoded and sent to the National Institute for Communicable Diseases (NICD) in Johannesburg, South Africa, together with its respective positive RDT cassette, for anti-malarial resistance marking [47]. An additional 10% of the negative RDTs were collected and sent to the NICD for quality assurance.

As malaria is a notifiable condition in South Africa, demographic and malaria case information collected at the malaria diagnosis and treatment initiation phase are reported on the Notifiable Medical Condition (NMC) form or mobile application. If reporting is paper-based, forms are collected by a MEP case investigator assigned to that healthcare facility, ideally within 24 h of diagnosis and delivered to the sub-district malaria office for data quality verification and data capture. Within 24–72 h of case notification, case investigators should visit the malaria patient’s household for in-depth case investigation to assess for the presence of malaria risk factors (e.g., last indoor residual insecticide spraying of that household or nearby mosquito vector breeding sites) and to conduct contact tracing and testing. During these case investigations, the household’s GPS location coordinates are recorded, and the case investigation form completed. The malaria treatment adherence and response information (including any side effects) is captured as part of the case investigation using Additional file 1: Tool S1. Once completed these forms are submitted to the sub-district malaria office for quality checking and electronic capture into the DHIS2.

### Data

#### Malaria case data

Malaria case data consisted of NMC, case investigation and treatment adherence and response forms as individual case records captured on paper or electronically, and downloaded from the DHIS2.

#### Geospatial data

The geospatial data consisted of four types. Firstly, each patient’s residential address and GPS coordinates were sourced from both MEP android tablets/handheld GPS devices and as recorded in DHIS2. Secondly, population settlement shapefile data were obtained from (a) the Ehlanzeni District Municipality, and (b) the open-source Global Administrative Areas website for South Africa [48]. Thirdly, a list of locality addresses of malaria cases were obtained from the Mpumalanga MEP office, curated and validated using Google Maps. Lastly, the modelled Facebook population of South Africa density raster was downloaded from The Humanitarian Data Exchange (HDX v1.52.1) [49].

#### Laboratory data

Parasite DNA was extracted from the RDTs and DBS using the Qiagen DNA mini extraction kit (Qiagen, Germany), according to the manufacturer’s instructions. When both the RDT and accompanying DBS were available, DNA was extracted from both sources in a single reaction. The extracted DNA was subjected to multiplex PCR to confirm *Plasmodium* species [50]. To assess possible decreases in lumefantrine susceptibility, samples containing only *P. falciparum* parasites were subjected to quantitative and conventional PCR, and endonuclease cleavage to detect polymorphisms at codons 72–76 of the chloroquine resistance transporter (*crt*) [51] and codon 86 of multi-drug resistance 1 (*mdr1*) genes [93]. Codons were classified as pure sensitive, pure mutant or mixed (both mutant and sensitive genotypes present in an individual patient’s sample). Genotyping assays were run in duplicate, with a third assay performed on any discordant results. When calculating overall prevalence of infections with mutant genotypes, codons with mixed genotypes were grouped with pure mutant codons. The copy number of the *mdr1* gene was assessed using quantitative PCR (qPCR), with primers, probes and qPCR cycling conditions previously described [52]. Every qPCR run contained three reference DNA samples from D10 and Fac8 clones, having an *mdr1* copy number of one and three respectively, as well as a no-template control. Assays were repeated if the threshold cycle values were greater than 35. For the assessment of artemisinin resistance, the propeller domain of the *k13* gene was amplified as previously described [53]. The amplified products were sent to Inqaba Biotechnologies (South Africa) for Sanger sequencing. Sequences were then aligned against a reference *k13* gene (XM_001350122.1) using a BLAST search and BioEdit Software [] to detect polymorphisms in 27 codons associated with delayed parasite clearance in South East Asia [54]. Molecular data were compiled monthly and shared with study investigators for further curation and analysis. Results were presented to the Mpumalanga MEP monthly and quarterly for them to take timely action with investigation and response in the event of any significant resistant mutations.

### Definition of metrics

#### Coverage

Four measures of coverage were used: (1) percentages of malaria cases with blood samples taken (RDT/DBS), (2) percentages of cases assigned a correct barcode (necessary for linkage of laboratory results to NMC data), (3) percentage of cases investigated and (4) percentages of investigated cases with GPS coordinates relative to all reported malaria notifications captured in the DHIS2.

#### Accuracy

Two measures of accuracy used were: (1) percentage of investigated cases with GPS coordinates falling within the study’s residential areas, and (2) percentages of notified cases with correctly formatted barcodes, calculated monthly and quarterly.

#### Linkage

This was measured using the percentage of cases with accurate barcodes linked to the NMC data, the molecular markers results data and accurate GPS coordinates at health facility, ward, locality, and household levels.

### Study procedures

The accuracy, coverage and ability to link the malaria notifications to the case investigation, laboratory data and drug report data was evaluated using monthly timelines. Data from DHIS2 was downloaded monthly from the malaria programme and shared with the project team for curation and analysis. A checklist was used to record the settings of each MEP GPS device, and their data was downloaded for further analysis. All data were securely downloaded, encrypted and transferred to the password-protected study computer for further compilation and analysis.

### Analysis

All data analyses were conducted using R programming language (versions 3.6 and 4.0) and Esri ArcGIS ArcMap (version 10.8). The analysis focused on data linkage, spatiotemporal trends in molecular marker and usability assessments. Coverage, accuracy and linkage metrics were used as units of analysis for temporal trends in the numbers of malaria cases reported, malaria cases investigated, the laboratory received samples, and post-treatment case investigation reports.

### Trend analysis

Monthly percentages were calculated using the monthly reported malaria case totals as the denominator. Time-series decomposition was used to evaluate for the non-stationarity of data and account for trend (t), seasonality (s) and random noise (r) [55]. Loess regression was used to obtain the optimum distribution and the 95% confidence margins of the trend (Additional file 1: Figures S2(a), S3(a), S4(a)). This time series was further decomposed to evaluate trend, seasonality and random errors using Sen’s slope and Mann–Kendall test [39, 56]. Seasonality was further explored using box plots.

### Molecular markers analysis

The classification of the 27 *k13* mutations after codon 400 assessed in this study was guided by the WHO [57, 58] and the Worldwide Antimalarial Resistance Network (WWARN) [54]. The 2020 WHO categories of 'validated', 'associated/candidate', 'not associated' or 'wild type' were used [58]. The 'wild type' parasite was renamed to 'sensitive' for further clarity. Mutations at codon 86 of the *mdr1* gene and codon 76 of the *crt* gene together with increases in *mdr1* gene copy number were assessed to determine susceptibility to lumefantrine. Parasites with the *mdr*86ASN and *crt*76LYS alleles but no increase in *mdr1* copy number were categorized as less susceptible (or tolerant) to lumefantrine, while those with an increased *mdr1* copy number considered lumefantrine resistant.

### Spatial and usability analysis

All shapefile data and residential coordinates from malaria cases were converted to the HartebeestHoek94 Datum coordinate system and projected to the Universal Transverse Mercator zone 32 [59]. Two draft maps were then drawn to display (1) thematic maps for the distribution of malaria cases by ward and (2) density maps of cases distributed by settlement within their ward boundaries. Twenty-four case investigators, with between one and 24 years experience working in Nkomazi sub-district, reviewed both maps to identify and label the Nkomazi wards (administrative level four). Feedback obtained from malaria case investigators was used to develop malaria case distribution maps and evaluate the shapefiles.

Thematic maps of the distribution of malaria cases by ward were produced using a spatial join tool linking GPS coordinates to the sub-district polygon. All cases falling in the same ward were summed, and an equal-interval scale and continuous colour ramp were used for displaying the distribution of cases by ward. Density maps of malaria cases per 1000 population were produced using kernel density estimation at 1 × 1 km and 0.5 × 0.5 km grids with a buffer around the sub-district polygon of 1 km and 0.5 km, respectively. The two grids were purposely selected for comparison. The Kernel density estimation used Quartic implementation as per the formula below:

$$Density=\frac{1}{{\left(radius\right)}^{2}}\sum_{i=1}^{n}\left[\frac{3}{\pi }\cdot {pop}_{i}{\left(1- {\left(\frac{{dist}_{i}}{radius}\right)}^{2}\right)}^{2}\right]$$

where, *i* = *1,…,n* are the input points. The sum of points was used if they were within the radius distance of the (x,y) location. *pop*_*i*_ is the population value of point i. *dist*_*i*_ is the distance between point i and the (x,y) location [40].

Feedback was obtained from the case investigators using semi-structured interviews to assess if the maps were well understood and whether the distribution of malaria cases corresponded with their local knowledge. A case-based orientation was used to optimize the maps to arrive at the most correct and easily understood versions.

Descriptive exploratory proximity analysis was further conducted on residential coordinates to ascertain the probability of these locations falling in the actual residential area (within 0.5 × 0.5 km) at a given time (t). Two types of analyses were used. Firstly, for identifying the progress of the accuracy and distribution of the malaria case residential coordinates over time, a time-series line using Loess regression was plotted, as were maps to assess the distribution of the coordinates. Secondly, the quadrats of the observed malaria cases at 0.5 km radius compared to expected cases were analysed by a Poisson process using the known malaria incidence and population settlement data to obtain likelihood ratios and Chi-square test. To assess the sparsity of malaria case residential data, average nearest neighbour analysis was used to explore for precision in the GPS coordinate dataset.

## Results

### Malaria notification, case investigation, drug adherence and response reports

**Fig. 2:**
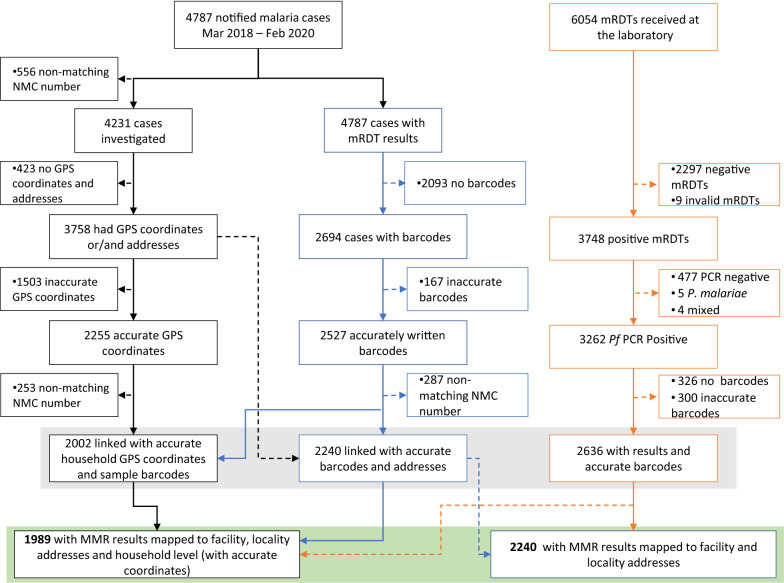
Making Data Map-worthy data flow chart. A chart showing data flow from the DHIS2 (consisting of malaria notification data captured on the notifiable medical condition (NMC) forms and case investigation data captured on case investigation forms), and molecular laboratory data on molecular markers of resistance from filter paper dried blood spots of RDT positive malaria patients. *MMR* molecular markers of resistance results, *RDT* malaria rapid diagnostic test, *Pf* *Plasmodium falciparum*)

From 1 March 2018 to 28 February 2020, 4787 malaria cases were notified in Nkomazi sub-district. All cases were definitively diagnosed using RDTs, with 98% (n = 4673) treated as outpatients. The probable source of infection for 73% (n = 3486) of the cases could be identified, with 96% of these classified as imported cases (i.e. source of infection outside South Africa). Of the 2531 cases with a reported date of diagnosis, the majority (80%) presented at health facilities within two days of symptom onset. Altogether, 78.5% (n = 3758) of cases were investigated and GPS coordinates captured (Fig. 2). Treatment adherence and response reports were introduced from January 2019 (Additional file 1: Figure S1), whereafter, of 2464 cases investigated, 61% (n = 1510) had the treatment adherence and response report completed. Overall, 72% (1793/2507) of cases were investigated within 24 h (and 75%, 81%, 87%, and 92% within 3, 7, 14 and 30 days, respectively).

Data linkage was performed at three levels, the first two at household level and the third at locality level. The first group linked case investigation data with accurate household GPS coordinates (n = 1053) and RDT barcodes (n = 2527), allowing 89% (n = 2002) of investigated cases to be linked to their molecular markers of resistance results. The second group included treatment adherence and response reports (Additional file 1: Tool S1), where only 50% (n = 1413, Additional file 1: Figure S1) of case investigations since its introduction in January 2019 could be linked to molecular markers of resistance results. The third group used GPS coordinates of locality addresses (n = 2255) as an anonymised proxy for residential coordinates, where 85% (n = 2240) of cases investigated could be linked to their individual molecular markers of resistance results (Fig. 2).

Of the 6054 RDTs received by the national laboratory, 61.6% (n = 3748) were reported as *P. falciparum* positive by the Mpumalanga MEP; the remainder were negative RDTs sent for quality control purposes. Parasite DNA was extracted, and PCR amplified from these positive RDTs and their corresponding filter paper dried blood spots (DBS), with 3340 (88%) found to be *P. falciparum* positive by PCR. Only samples with *P. falciparum* mono-infections [98% (n = 3262)] were assessed for molecular markers of drug resistance, of which 80.8% (n = 2636) had barcodes for linkage.

**Fig. 3:**
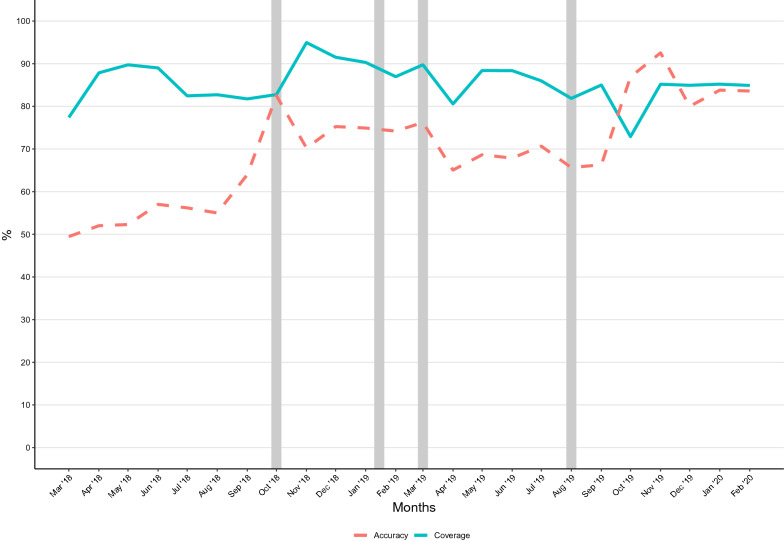
GPS coordinate coverage and accuracy. The coverage and accuracy of the GPS coordinates were assessed over the two-year study period (March 2018–February 2020). The grey bars indicate when training was conducted

**Fig. 4:**
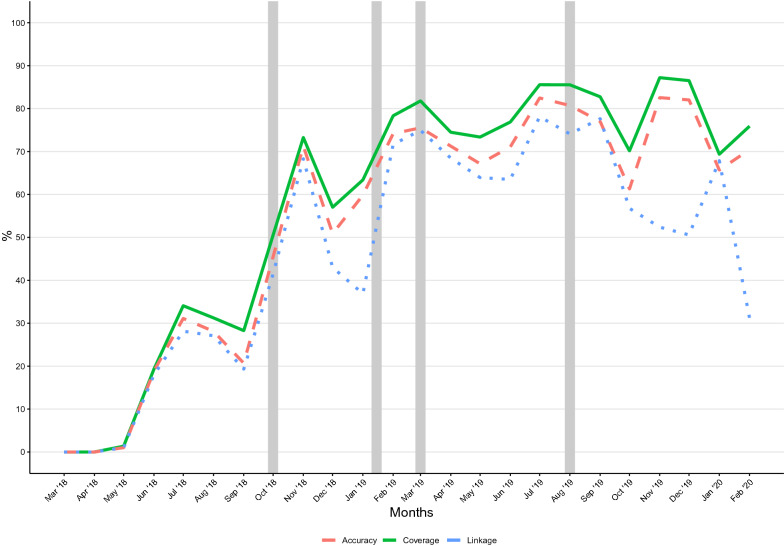
Barcode coverage, accuracy and linkage. The coverage, accuracy and linkage of the barcodes were assessed over the two-year study period (March 2018–February 2020). The grey bars show when training was conducted

Linkage of the molecular markers of resistance results and case notification data increased to 72% (95% CI 60—82%) at the end of the second quarter of 2019 before dropping to 47% (95% CI 38–60%) in quarter one of 2020 (Additional file 1: Figure S6). Molecular marker results could be linked to 99% (n = 1989) of the notified cases with accurate barcodes and residential coordinates, and 2240 cases with accurate locality addresses and barcodes (Figs. 3 and 4).

### Temporal trends of the selected surveillance metrics

**Fig. 5:**
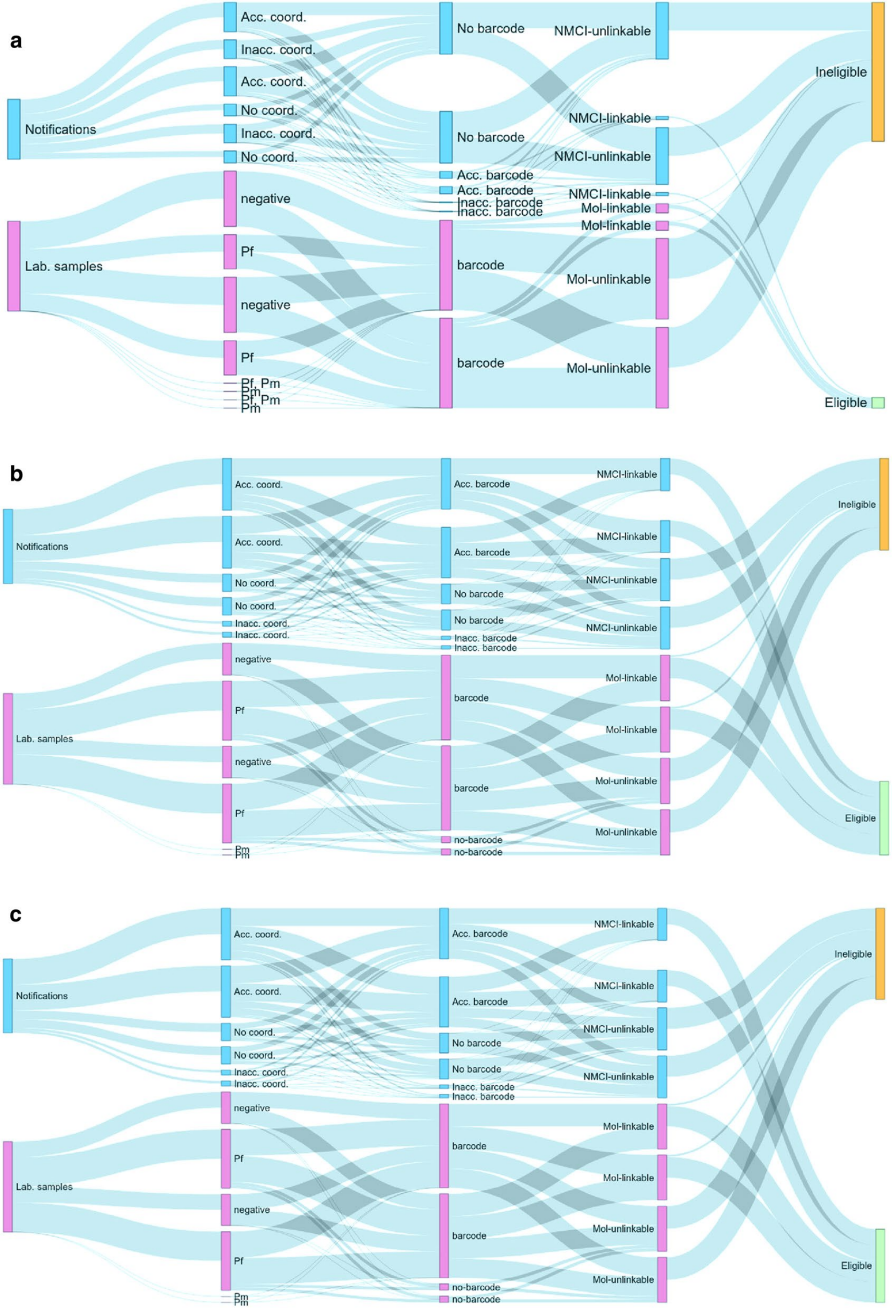
The longitudinal flow of data over the study period (March 2018–February 2020). Making Data Map-worthy (MDM) data flow over time from malaria case notification and laboratory data. The coloured bars show the totals, while the flows in grey illustrate the proportions of data that corresponded to the destination bar for the period. Over time, coverage, accuracy and linkage increased, illustrated by increased sizes of the corresponding bars for (**a**) March–August 2018, (**b**) September 2018–June 2019 and (**c**) July 2019–February 2020. (Acc. coord.: accurate residential coordinates, Inacc. Coord.: inaccurate residential coordinates, *NMCI* notifiable medical condition notification and case investigation data linkable/unlinkable, *Mol* molecular marker of resistance data linkable/unlinkable, *Pf Plasmodium falciparum*, *Pm Plasmodium malariae*

As shown in Fig. 2 (overall) and Fig. 5 (longitudinal analysis by semester), linkage data increased from 12% at baseline to 54% in the final quarter. Barcoded case notification forms increased from 38 to 97% overall, while of RDT samples received at the NICD laboratory, those barcoded increased from 19 to 85% (Fig. 4). The household GPS coordinate accuracy increased from 48 to 76% over the study period (Fig. 3).

### GPS coordinate coverage and accuracy

Although the proportion of households with GPS coordinates (“coverage”) remained high throughout the study, spikes in coverage that corresponded with the months after on-site training were noted except following the third training. However, the accuracy of these coordinates increased from 48% at baseline to 89% in November 2019, with high levels of accuracy sustained until February 2020 (Fig. 3 and Additional file 1: Figure S3).

### Barcode coverage and accuracy

Over the course of the study, there was a steady increase in the percentage of accurately barcoded samples. Coverage and accuracy increased from an average of 5% in the first quarter to 80% in the last quarter, again with peaks in the months after on-site visits and training, except for the third training (Figs. 4 and Additional file 1: Figure S4). Over the two study years, there was a steady increase in barcode recording accuracy, reaching 75% (95% CI 64–85%) by the last quarter of 2019; however, this dropped to 64% (95% CI 59–85%) in the first quarter of 2020 (Additional file 1: Figure S5).

### Spatial analysis and semi-structured evaluation of the spatial data

**Fig. 6:**
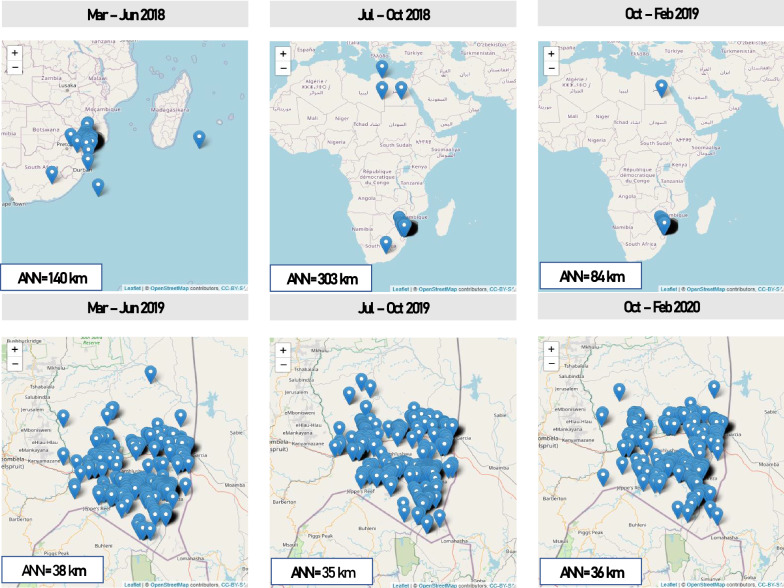
GPS coordinates of the malaria case residential locations collected during case investigation by quarter (2018–2020). Distribution of GPS coordinates in the six quarters evaluated. The top three maps show some highly dispersed coordinates far away from the study area compared to the subsequent period shown in the bottom three maps. Training using SOPs in Additional file 1: Tool S2 were conducted in October 2018, January, March and August 2019

Widely dispersed household coordinates were obtained in the first three-quarters of the study, including positive and negative coordinates (hence some coordinates in the northern hemisphere or ocean), as illustrated in Fig. 6. Twenty-eight GPS collection devices used by Mpumalanga MEP case investigators were assessed. All 19 Android device GPS capturing applications had degrees and decimal minutes (DDD° MM.MM'), while 5/9 handheld Garmin eTrex-10 devices had decimal degrees and decimal minutes (DDD.DDDD^o^) and the remaining four in the format of degrees, minutes and seconds (DDD° MM' SS.S"). Standard operating procedures (Additional file 1: Tool S2) were developed, which included how to set devices to decimal degrees, and four workshops were conducted (November 2018, January, March and July 2019) to train case investigators on best practices for the collection of GPS data.

Overall, improvement in the accuracy and precision of coordinates was observed over the study period. The average nearest neighbour distance decreased from 330 km in the second quarter to 35 km by the fifth quarter. The proportion of residential coordinates of a given malaria case falling within the 0.5 km × 0.5 km area rose from 15% (95% CI 4–48%) in the first quarter to 88% (95% CI 72–96%) by the 5th quarter.

Of the maps generated (Additional file 1: Figure S7a), the density map of the distribution of cases by 0.5 × 0.5 km grid was preferred by the 24 MEP staff interviewed. Problems identified in the thematic map (Additional file 1: Figure S7b) included colouring of the whole ward polygon while malaria cases are clustered only in certain areas within the wards (other areas are largely unoccupied farmlands or nature reserves), so not residing equally throughout the ward as shown in the choropleth ward map. In addition, non-familiarity with the ward demarcations was demonstrated by case investigators failing to label the respective wards (5/38), with duplicate labelling (7/38) and misplaced labels with no consensus (12/38) reported.

### Molecular markers of drug resistance

Of the 3748 malaria-positive RDTs, 13% (n = 477) were malaria negative by PCR, five were pure *Plasmodium malariae* and four were mixed infections (*P. malariae* and *P. falciparum*). Of the 2297 RDTs reported as negative by the Mpumalanga MEP, 2% (53) were found to be false negatives by PCR. Of the false-negative RDTs analysed, 96% (51/53) were found to be pure *P. falciparum* infections, with the remaining 4% (2/53) pure *P. malariae* infections by PCR.

The propeller domain of the *k13* gene was successfully amplified and sequenced from 73% (2385/3262) of the PCR positive falciparum samples (Table 1). All sequenced samples were wildtype at the 27 *k13* single nucleotide polymorphisms (SNPs) known to be associated with artemisinin resistance (delayed parasite clearance).

**Table 1:** Summary of molecular markers of artemisinin and lumefantrine “resistance”

	Artemisinin	Lumefantrine	
Marker name	*k13*	*Pf mdr186*	*Pf crtK76T*
Samples assayed (n)	2385	2812	2122
Wild type	2385 (100%)	2803 (99.7%)	2121 (99.9%)
Mutant	0(0%)	9 (0.3%)	0 (0%)
Mixed	0(0%)	0 (0%)	1 (0.1%)

Prevalence of *k13*, *mdr186* and *crt76* mutations in individual patients with *P. falciparum* infections, Nkomazi Sub-District, Mpumalanga (March 2018–Feb 2020). Markers showing sensitive parasites include the wild type-*k13*, mutant-*mdr186*, and mutant/mixed *crtK76T* and potentially reduced susceptibility (or tolerant) markers with wild type-*mdr186* and *crtK76T*

**Fig. 7:**
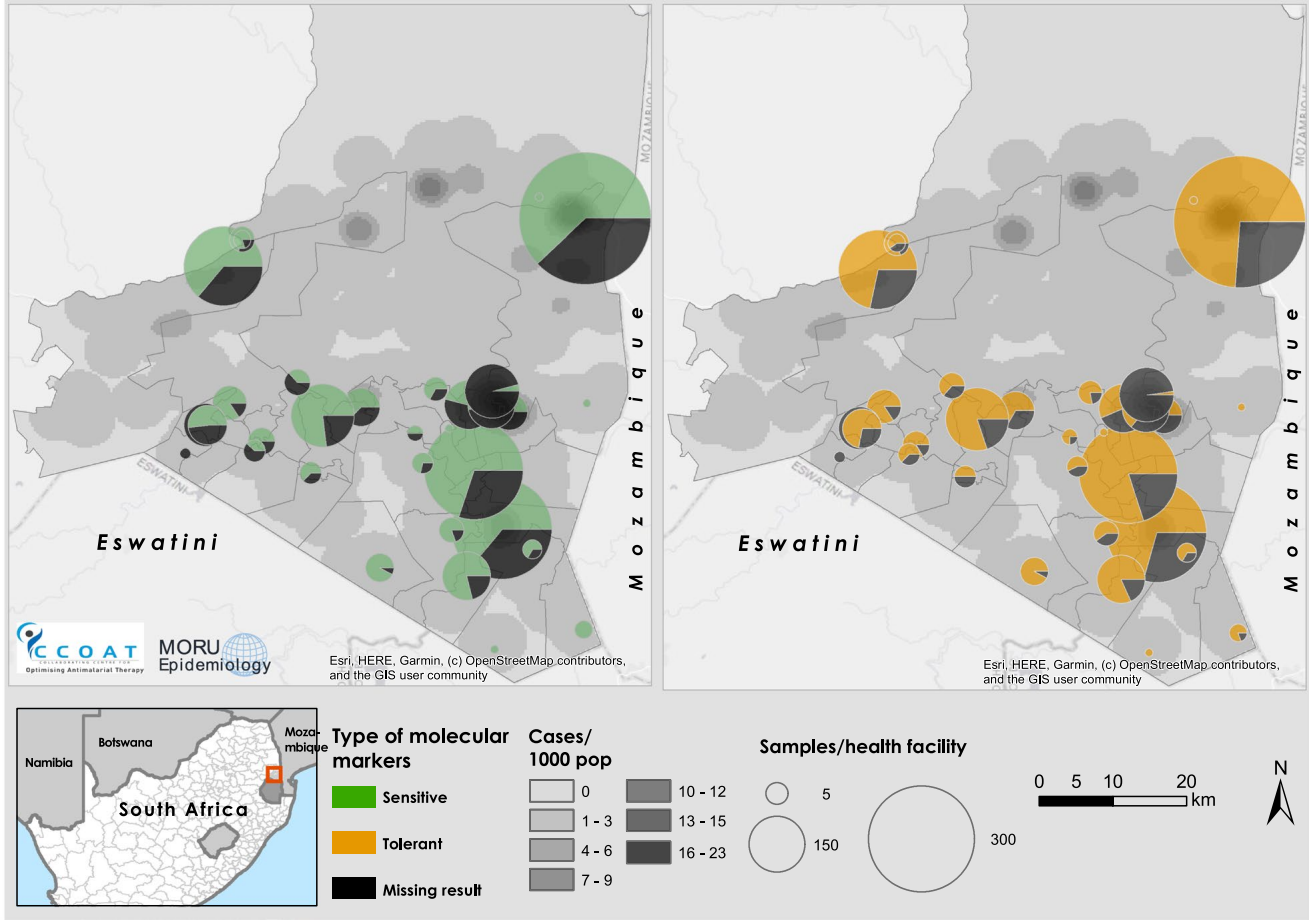
Distribution of confirmed malaria cases and molecular markers of artemisinin and lumefantrine drug “resistance” in Nkomazi sub-district, Mpumalanga (March 2018–February 2020). Distribution of *P. falciparum* malaria cases by 5 × 5 km grid, artemisinin *Plasmodium falciparum* k13 (left) and lumefantrine (right) mdr186ASN/crt76LYS molecular markers of “resistance”, denoted by their susceptibility

Almost all the samples in which the *mdr186* and *crt*76 SNPs could be assessed carried wild type *mdr186*ASN (99.7%, 2803/2812) and *crt*76LYS (99.9%, 2121/2122) alleles, respectively (Table 1). No increase in copy number was observed in the 1503 isolates assessed for *mdr1* copy number. Thus, these samples were classified as potentially having reduced susceptibility (or tolerance) to lumefantrine, but not resistance, as shown in Fig. 7.

## Discussion

Over the course of this study, data was curated from 4787 notified malaria cases and 55.1% of these cases were linked to their individual anti-malarial drug resistance profiles and residential localities in Nkomazi sub-District, a pre-elimination area in Mpumalanga, South Africa. This pilot evaluation used an iterative framework, termed ‘Making Data Map-worthy’. This is the first study utilising routine malaria surveillance data individually linked to molecular surveillance data to create near-real-time maps of anti-malarial drug resistance. The evidence generated by this pilot exemplifies the WHO recommendation to transform surveillance into a core intervention [23]. While most evaluations of molecular markers of artemisinin and partner drug resistance are from clinical trials, routine surveillance has the potential to facilitate the early detection of anti-malarial drug resistance in areas in which clinical trials are not feasible, such as low transmission intensity areas and areas where most malaria occurs in highly mobile and migrant populations [22, 60]. Although the WHO recommends iDES for low malaria transmission settings, there is little evidence of its feasibility in resource-constrained malaria programmes with thousands of malaria cases, particularly if most infections are among mobile and migrant populations. The findings from this study present a possible solution by allowing malaria programmes in such settings to target where and when resource-intensive confirmatory investigations and additional transmission-blocking interventions are needed [60].

Routine malaria surveillance data were collected and assessed over two years to map any spatiotemporal changes in anti-malarial drug resistance molecular markers. A user-centred feedback approach helped to assess data quality and incorporate improvement activities into data collection, analysis and map creation. Understanding malaria programmatic needs to support public health decision-making using an integrative approach, such as user-feedback and co-creation, has previously been shown to assist in the take-up and sustainability of new interventions, especially those that involve new technology adoption [35]. The greatest improvements in the surveillance metrics studied were generally observed following on-site supervision, and were sustained at a moderately high level for seven months after the last on-site supervision visit. Since there are no proposed analysis frameworks for evaluating routine location data from cohorts of infectious diseases patients, metrics were adapted from the latest systematic review and the WHO's Data Quality Review (DQR) framework, an expert proposed framework designed only for the assessment of facility-based data [61, 62]. Malaria surveillance data include off-facility activities such as case investigation home visits to assess treatment adherence and response, while seeking any mosquito vector risk factors.

A 0.5 × 0.5 km malaria density map provided the most user-friendly representation of the distribution of malaria. This finding challenges the most prevalent malaria map designs, namely ward level thematic maps of case distribution, which show that geographical or political boundaries demarcate cases. Density and other modelled maps can show disease distribution beyond uninterrupted land borders, which relates better to how infectious diseases, such as malaria spread. Another advantage of the density maps in this area was the avoidance of large areas used for plantations and a national game reserve that lacked human settlements for malaria transmission. Density maps displaying cases in a continuous land surface require modelling of the incidence and other key covariates that determine the distribution of cases. This study used the latest available human settlement data and feedback from 24 local malaria case investigators to validate the correct malaria case distribution in their areas.

While only 55.1% of all reported malaria cases could be linked, overall, the iterative analysis, training and feedback improved the precision of collected GPS data from 15 to 88% within 0.5 × 0.5 km grid squares. With 89% (n = 2002) of investigated cases having accurate GPS location linked to their individual molecular marker of resistance results, it would be possible to target the correct geolocation of a given case for further investigation and prompt response within that community, should any molecular marker/s of concern be identified.

Almost 45% of individual malaria cases and molecular data could not be linked in this study. To achieve optimum linkage and data curation might only be possible with the iterative analysis of data, identification of gaps and implementation of collaborative surveillance strengthening activities, in order to improve the data collection, capture, analysis and reporting cycle. Although this may be perceived as resource-intensive and challenging to implement in a low resource setting, such an investment is essential for all malaria surveillance objectives to be achieved, not just for promptly detecting, locating and responding to any emerging anti-malarial drug resistance. To avoid straining the already stretched health system, further development of assay methods is needed to obtain an adequate yield from RDTs alone, as filter paper DBS requirements could potentially limit the scalability of this approach. Several studies have proposed the use of RDTs for parasite DNA extraction as a useful alternative to the current methods; however, its applicability at field level is yet to be established [64–67]. Even for next generation sequencing, using DBS is recommended as the DNA obtained from RDTs is generally insufficient and of poor quality [68]. Pooling individual patient DBS samples before performing a genomic analysis has been proven useful for low and high malaria transmission settings [69]; however, no such pooling of samples has been reported yet for RDTs. Morris et al. 2013 noted that when RDTs were used alone, the DNA yield was much lower, allowing for only a “one-shot operation” with no possibility of DNA re-extraction [65]. Thus, each case’s RDT and blood spot were used together in a single reaction to increase the parasite density, if both were present. The DNA yield for RDT vs DBS was not quantified or compared in this study.

Although numerous human and system errors were identified and corrected, especially in the first and second quarters, a significant proportion of the cases could not be linked to their resistance profile or locality. The inability to follow-up patients, particularly those among the highly mobile migrant populations, played a significant role in the low number of household coordinates collected. Although many of the migrant cases presented at local healthcare clinics, most could not be followed up to geolocate their residential addresses/ward or assess treatment adherence and response. Undocumented migrants may be more likely to provide inaccurate contact details for the notification form, and many transit rapidly through endemic areas to reach major cities in non-endemic areas.

Although the proportion of filter paper DBS samples submitted with positive RDTs increased over the study period to 92% in the final quarter, the quality of the DBS collected remained suboptimal. Only 61% of the collected DBS passed the internal quality control screening, in terms of sufficient blood volume and storage conditions to be entered into the laboratory workflow. Despite numerous training rounds, the health facility staff persisted in collecting very low blood volume (less than 10 µl) DBS. These low blood volumes decreased the efficiency of both the DNA extraction and downstream PCR analyses, particularly in infections with low parasite densities. It has been shown that DBS with at least 50 µl of blood are essential for molecular assays that include next-generation sequencing [70]. More intensive in-person training would be required to improve and sustain progress.

A small proportion (2.2%) of negative RDTs were malaria positive by PCR. This finding could be due to patients with infections that have parasites loads below the detection limit of the RDT (200 parasites per µl blood), but within the detection limit of the more sensitive PCR assay (20 parasites per µl blood). Other possible explanations include inadequate storage conditions of the RDT and/or DBS or the incorrect use of the RDT (addition of too little blood or too much Lysis buffer or reading before the recommended time). Preliminary investigations suggest these false negatives were not due histidine-rich protein 2 (*hrp*2) deletions; available evidence suggests *hrp2* mutations are currently rare in southern African [71]. However, ongoing systematic testing is required to exclude *hrp*2 deletions in this region.

Fortunately, neither 'validated' or 'associated/candidate' *k13* mutations associated with artemisinin resistance were found this study [54, 57, 58]. However, the strong selection for the *mdr*86ASN and *crt*76LYS wildtype alleles may indicate some lumefantrine tolerance. A study in Uganda found an increased relative risk of treatment failure (PCR-adjusted) associated with the presence of the *mdr*86ASN allele [72]. Venkatesan et al. found a slight increase in recrudescence and reinfection for parasites carrying *pfcrt*76 and *mdr*86ASN alleles following AL treatment [73]. However, no other African studies have demonstrated an increased therapeutic failure rate when these alleles are present [74–76]. In Asia, clinical failure rates have been linked to the increase in *mdr1* copy number as compared to the *mdr*86ASN alleles [77]. The increase in *mdr1* copy number has rarely been reported in Africa [52, 78], and this study did not observe any such increase.

This study highlights the need for continued rigorous surveillance, particularly in light of multiple reports of the independent emergence of *k13* resistance markers in Central [10], East [11–13], and West African countries [13, 14]. Despite the absence of validated *k13* artemisinin resistance mutations in southern Africa, the decline of ACT clinical efficacy below the WHO threshold of 90% observed in nearby Angola in 2013 and 2015 (Zaire Province) and 2019 (Lunda Sul Province) [79] is of some concern [80], although consecutive studies in 2017 and 2019 showed adequate parasite clearance rates [79, 81]. The extreme AL drug pressure in sub-Saharan Africa, delayed parasite clearance following AL treatment in Rwanda, and the emergence of clinical artemisinin resistance in Uganda calls for strengthening resistance surveillance across Africa. Such activities will inform efficient targeting of transmission blocking activities (including SLD primaquine and foci clearing) and further investigation of parasite clearance rates and ACT therapeutic efficacy, with prompt changes to treatment policy[17] should treatment failure rates exceed acceptable limits (currently 10%) [57].

Some of the challenges and limitations often seen with the use of routine surveillance data were also encountered in this study, including limited data availability, multiple information/reporting systems and relatively high staff turnover rates. During the course of the study, DHIS2 was being rolled out and updated, while malaria cases notified before the generic notifiable medical condition (NMC) system was introduced in January 2019 were entered in a provincial MS Access-based Malaria Health Information System. The transition between these overlapping systems might have affected the data capturing cycle and impaired harmonization of the two datasets, potentially impacting on data quality. During this transition period, malaria cases could be notified using paper notification forms (later captured into the DHIS2) or on one of two mobile-phone-based systems, a malaria case short messaging service and NMC mobile applications[82]. Ideally, these two systems should feed into the DHIS2 system and remove duplication; however, these two mobile-phone-based databases could not be accessed for confirmation. Insufficient staff and resources may also hamper data quality. During the two years of this study, some of the staff from a collaborative non-governmental organization (who comprise more than half of the malaria case investigators) had to stop working for a few months whilst waiting for the renewal of funding, and this interrupted their surveillance activities. Other studies have also documented similar challenges leading to inconsistencies and inaccuracies in health information systems [83–85]. Such challenges limit the usage of routine data in decision-support systems, especially in low-resource settings. Consequently, countries rely on population-level health surveys, which remain costly, outdated and irregular; for instance, the WHO world malaria report still relies on modelling data due to incompleteness and inconsistencies in the malaria routine reporting system [1, 3, 86–88]. Other factors such as climate, altitude, vectors, or the existing malaria interventions that have been shown to affect the distribution of malaria cases in previous studies were not explored [33, 89], given the focus of this study.

Routine near-real-time mapping of molecular markers of anti-malarial drug resistance data to the healthcare facility, locality and patient household levels offers malaria programmes rapid and efficient monitoring of spatiotemporal changes in anti-malarial drug resistance profiles. By improving the facility and population-based routine surveillance systems as shown in this study, malaria programmes can identify areas of concern requiring further investigation and conduct targeted therapeutic efficacy trials and transmission limiting activities, hence allocating their resources strategically. This might however be too costly for high transmission settings. Here, molecular markers from a representative sample of malaria cases from a range of health care facilities with optimal geographic and epidemiological coverage could be used to strengthen resistance surveillance and inform programmes of areas where further investigation should be conducted. Such sentinel sites could be linked to a centralized national or regional laboratory, reducing investments and running costs. Low and moderate transmission settings have started implementing centralized genomic surveillance. For instance, Haiti [90], Honduras [91] and South Africa [28] provide examples of national molecular surveillance, while the GenRe-Mekong study provides a model for regional surveillance [92]. However, a feasibility study and cost-effectiveness analysis may be needed to inform the relevance of such a system in high malaria transmission settings. Pragmatic and innovative approaches such as co-design can enable precision mapping, contextualization of analyses and meeting of malaria MEP needs.

Although linking individual patient information and the molecular markers might not directly benefit the patient, the molecular results may be of value in case the patient returns to a healthcare facility with recurrent malaria, as the linked molecular marker data will help differentiate anti-malarial resistance from other causes of treatment failure and thus inform re-treatment strategy. While enhancing the quality of routine data can be a daunting task, identifying, monitoring and improving important surveillance metrics and indicators by MEPs is considered critical to both evaluating progress and achieving malaria elimination targets. This is consistent with the WHO recommendation that surveillance is a core intervention to achieve elimination. Countries that have eliminated malaria have established strong information systems and maintained them to prevent the re-establishment of the disease [23]. Sustainability can be facilitated by researchers and MEPs working collaboratively to develop tools and resources for efficient training and regular supervision that can be cascaded to reach all relevant MEP staff.

## Conclusions

In low malaria transmission settings in sub-Saharan Africa, near-real-time fine-scale mapping of molecular markers of anti-malarial drug resistance can assist in rapidly and efficiently monitoring anti-malarial drug resistance and identifying areas requiring further investigations and interventions. However, the sustainability of such a strategy requires regular training, close supervision and strong programmatic support. More innovation and research are needed to explore more cost-effective strategies for anti-malarial resistance surveillance systems given current resource constraints, such as sampling at representative sentinel health facilities strategies versus comprehensive sampling, linkage at individual versus health facility levels, particularly in moderate and high transmission settings. The methods piloted, and lessons learnt in this study could inform scale-up to provincial, national and regional malaria control/elimination programme levels in low- and middle-income countries and may be relevant for other antimicrobial resistance surveillance.

## Supplementary Information

**Additional file 1:** **Figure S1.** Data flow and linkage for notified malaria cases and case investigation reports on drug adherence and response. **Figure S2.** GPS coordinates’ coverage trend over the two-year study period (2018–2020). **Figure S3.** Accuracy trend of malaria case residential coordinates collected over the two-year study period (2018–2020). **Figure S4.** Percentage of mRDTs barcoded over the two-year study period (2018–2020). **Figure S5.** Barcode accuracy trend over the two-year study period (2018–2020). **Figure S6.** Linkage of the patients’ and antimalarial resistance data over the two-year study period (2018–2020). **Figure S7.** Three different types of shapefiles evaluated for the study area. **Tool S1.** GPS tools for training malaria case investigators. **Tool S1A.** Training guide for trainers. **Tool S1B.** Standard operating procedures for eTrex 10 GPS device (adapted from Health Geolab Collaborative, 2018).

## Data Availability

The datasets generated and/or analysed during the current study are available at the WWARN Tracking Resistance website (https://www.wwarn.org/tracking-resistance/artemisinin-molecular-surveyor).
