# Supplementary material for: Making data map-worthy—enhancing routine malaria data to support surveillance and mapping of Plasmodium falciparum anti-malarial resistance in a pre-elimination sub-Saharan African setting: a molecular and spatiotemporal epidemiology study
Source: Malar J. 2022 Jun 29;21:207. doi: 10.1186/s12936-022-04224-4 (PMC9244181; doi:10.1186/s12936-022-04224-4)
Supplement: Supplementary file 1 — Additional file 1: Figure S1. Data flow and linkage for notified malaria cases and case investigation reports on drug adherence and response. Figure S2. GPS coordinates’ coverage trend over the two-year study period (2018–2020). Figure S3. Accuracy trend of malaria case residential coordinates collected over the two-year study period (2018–2020). Figure S4. Percentage of mRDTs barcoded over the two-year study period (2018–2020). Figure S5. Barcode accuracy trend over the two-year study period (2018–2020). Figure S6. Linkage of the patients’ and antimalarial resistance data over the two-year study period (2018–2020). Figure S7. Three different types of shapefiles evaluated for the study area. Tool S1. GPS tools for training malaria case investigators. Tool S1A. Training guide for trainers. Tool S1B. Standard operating procedures for eTrex 10 GPS device (adapted from Health Geolab Collaborative, 2018). [file 12936_2022_4224_MOESM1_ESM.docx]

**Additional file 1**

**Making data map-worthy—enhancing routine malaria data to support surveillance and mapping of *Plasmodium falciparum* anti-malarial resistance in a pre-elimination sub-Saharan African setting: a molecular and spatiotemporal epidemiology study**

Frank M. Kagoro ^1 2,3,4,5^, Elizabeth Allen ^1,3,4^, Aaron Mabuza ^1,3^, Lesley Workman ^1,3,4^, Ray Magagula ^6^, Gerdalize Kok ^6^, Craig Davies^7^, Gillian Malatje ^6^, Philippe J. Guérin ^3,4,5^, Mehul Dhorda ^2,4,5^, Richard J. Maude ^2,5,8,9^, Jaishree Raman^10,11,12#^, Karen I. Barnes ^1,3,4,#,*^

**First author**

Dr Frank M. Kagoro, M.D., PgD HCTMC, MSc,

[kgrfra001@myuct.ac.za](mailto:kgrfra001@myuct.ac.za)

PhD Fellow | University of Cape Town, South Africa

Research Physician | Epidemiology Department, Mahidol-Oxford Tropical Medicine Research Unit, Faculty of Tropical Medicine, Mahidol University

Research Physician | Infectious Diseases Data Observatory (IDDO), Centre of Tropical Medicine and Global Health, University of Oxford

**Corresponding Author:**

**Prof Karen I. Barnes**

[karen.barnes@uct.ac.za](mailto:karen.barnes@uct.ac.za)

Professor: Division of Clinical Pharmacology| University of Cape Town (UCT)

Founding Director| MRC Collaborating Centre for Optimising Antimalarial Therapy (CCOAT)

Director: Pharmacology| WorldWide Antimalarial Resistance Network (WWARN)

# These authors contributed equally

*Corresponding author

Table of Contents

[**Figure S1: Data flow and linkage for notified malaria cases and case investigation reports on drug adherence and response** 3](#_Toc94629059)

[**Figure S2. GPS coordinates’ coverage trend over the two-year study period (2018-2020)** 4](#_Toc94629060)

[**Figure S3. Accuracy trend of malaria case residential coordinates collected over the two-year study period (2018-2020)** 4](#_Toc94629061)

[**Figure S4. Percentage of mRDTs barcoded over the two-year study period (2018-2020)** 5](#_Toc94629062)

[**Figure S5. Barcode accuracy trend over the two-year study period (2018-2020)** 6](#_Toc94629063)

[**Figure S6. Linkage of the patients’ and antimalarial resistance data over the two-year study period (2018-2020)** 7](#_Toc94629064)

[**Figure S7: Three different types of shapefiles evaluated for the study area** 8](#_Toc94629065)

[**Tool S1. GPS tools for training malaria case investigators** 9](#_Toc94629066)

[Tool S1A: Training guide for trainers 9](#_Toc94629067)

[Tool S1B: Standard operating procedures for eTrex 10 GPS device (adapted from Health Geolab Collaborative, 2018). 13](#_Toc94629068)

## **Fig. S1 Data flow and linkage for notified malaria cases and case investigation reports on drug adherence and response**

| **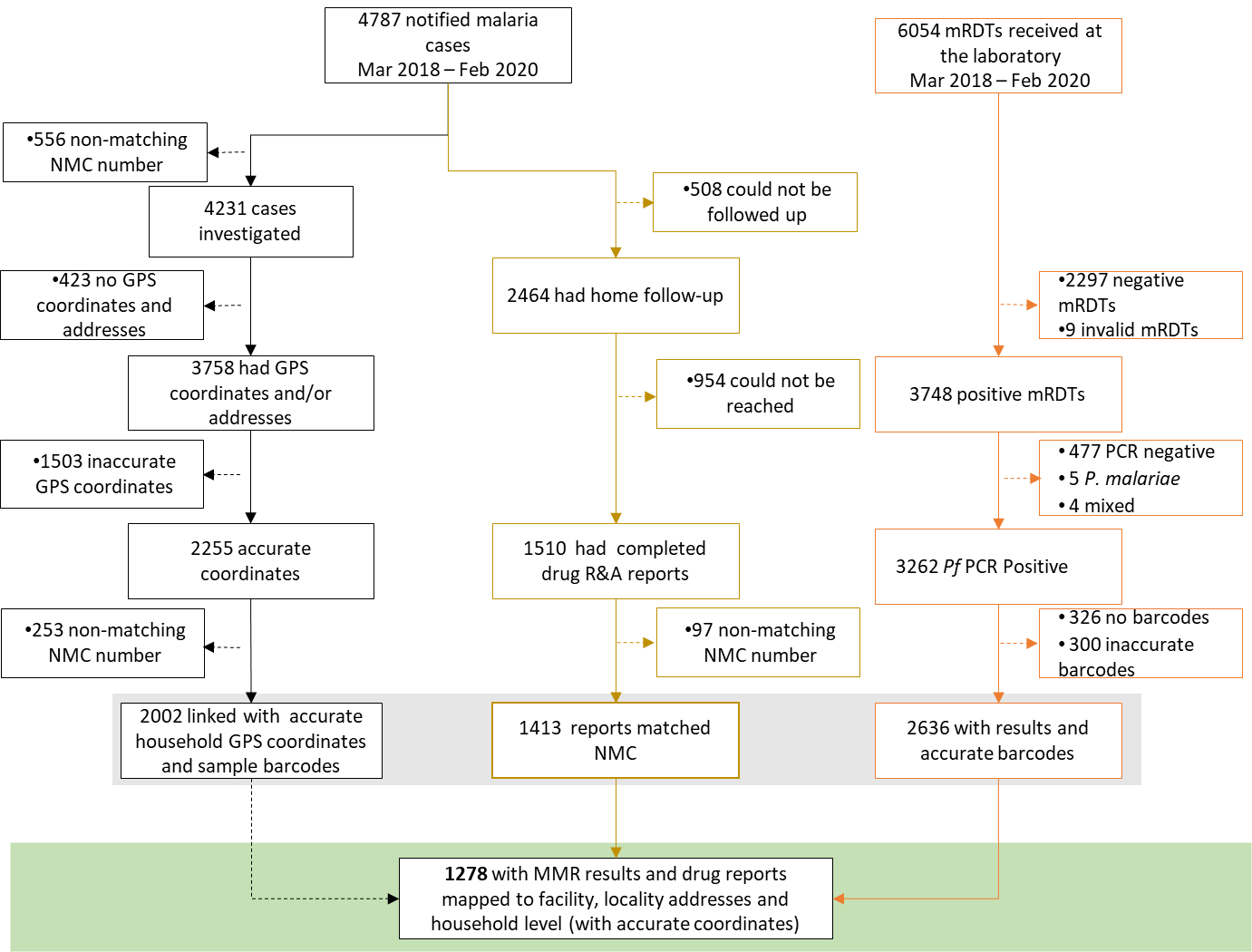** |
| --- |
| *A chart showing data flow from the DHIS2 (including clinical data of malaria notifications and tested samples captured by NMC and case investigation data captured on case investigation forms) and molecular laboratory data. Case investigation reports on drug adherence and response were only available from January 2019 when home follow-up for adherence and response was initiated. (MMR=Molecular markers of resistance results, mRDT=malaria rapid diagnostic test and P.f = Plasmodium falciparum)* |

## **Fig. S2 GPS coordinates’ coverage trend over the two-year study period (2018-2020)**

| 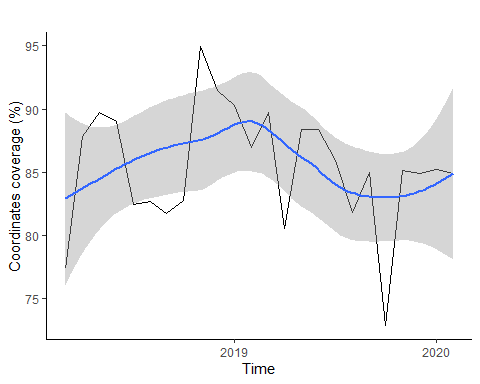a) | 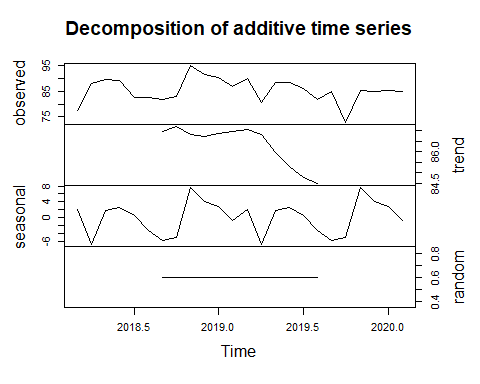b) |
| --- | --- |
| *a) a plot of mean coverage of coordinates, with 95% confidence intervals b) A decomposed time series of cases. The series was investigated with residential coordinates recorded over the two-year study period and accounted for the effect of random errors. The coverage had stationarity at 88% from 2018 until the second quarter of 2019, when it dropped to 85%.* | |

## **Fig. S3 Accuracy trend of malaria case residential coordinates collected over the two-year study period (2018-2020)**

| 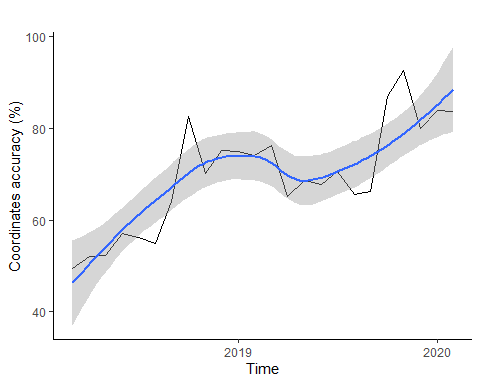a) | 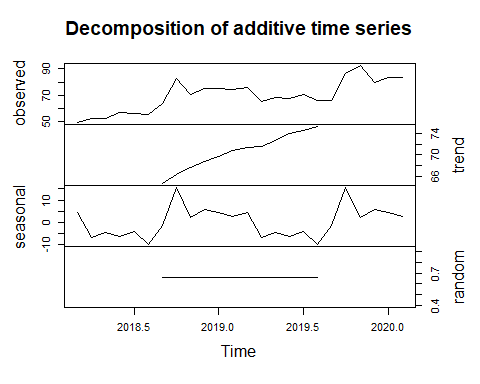b) |
| --- | --- |
| *a) Chart showing the trend for coordinate accuracy, with 95% confidence intervals, and b)a decomposed time series over the two-year study period. Accuracy ranged 48% (95%CI: 39 - 56%) at baseline to 89% (95% CI: 80 - 98%) by the first quarter of 2020 (Fig. S3. After accounting for the effect of random errors, there was a 1.4% (95% CI: 0.9 – 1.9 %) average increment in accuracy every month for two years (p≤ 0.001)****a*** | |

## **Fig. S4 Percentage of RDTs barcoded over the two-year study period (2018-2020)**

| 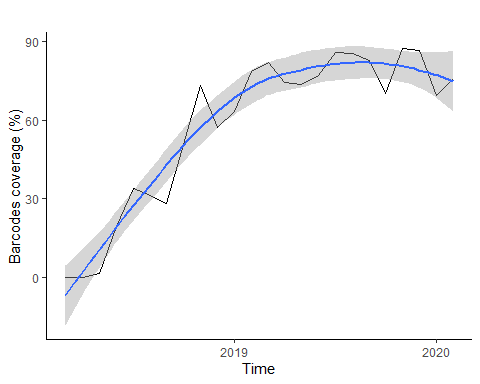 | 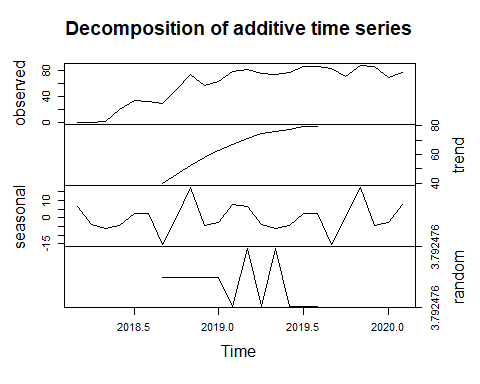 |
| --- | --- |
| *a) Chart showing the trend of barcode coverage and its 95% confidence interval, and b) a decomposed time series for two years. After accounting for the effect of random errors in the exploratory analysis, the coverage increased steadily throughout the two years of the study, with an average increment of 4.6% (95% CI: 2.2 – 4. 7%, p < 0.0001) per month.* | |

## **Fig. S5 Barcode accuracy trend over the two-year study period (2018-2020)**

| 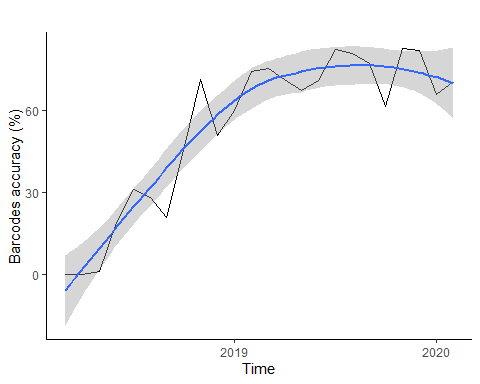 | 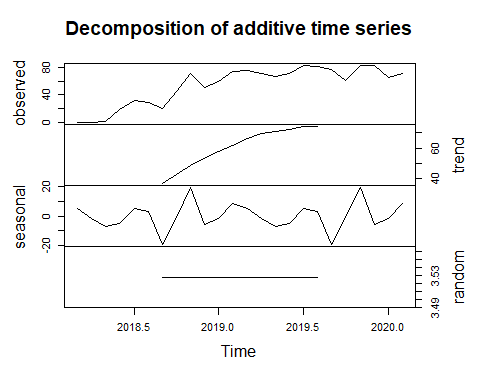 |
| --- | --- |
| *Figures showing a) the trend of barcode accuracy and its 95% confidence interval for two years, and b) its decomposed time series. After accounting for the effect of random errors, the accuracy increased steadily throughout with a 4.3% (2.1– 4.5 %) average increment per month (p < 0.0001).* | |

## **Fig. S6 Linkage of the patients’ and antimalarial resistance data over the two-year study period (2018-2020)**

In an exploratory analysis, after accounting for the effect of random errors, the linkage ability increased with a 3.1% (1.2– 4.1 %) average increment in linkage per month (p<0.0001) over the two-year study period until the 1st quarter of 2019 but plateaued after that (Fig. S6).

| 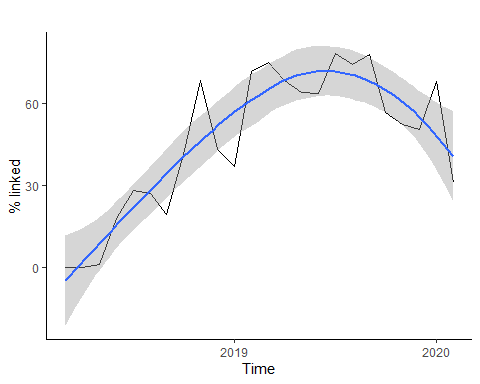 | 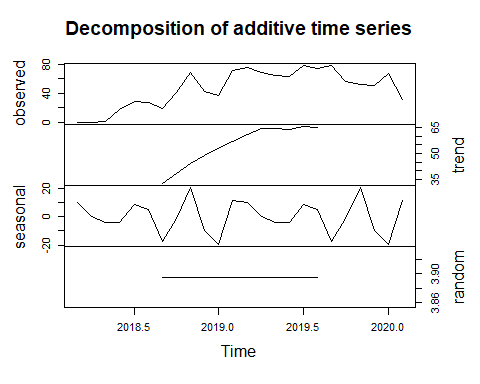 |
| --- | --- |
| ***Figures showing a) the trend of*** *data linkage, and its 95% confidence interval for two years, and b) its decomposition time series. after accounting for the effect of random errors, the linkage ability increased with a 3.1% (1.2– 4.1 %) average increment in linkage per month (p<0.0001) over the two-year study period until the 1st quarter of 2019 but plateaued after that (Fig. S6)* | |

## **Fig. S7 Three different types of shapefiles evaluated for the study area**

| 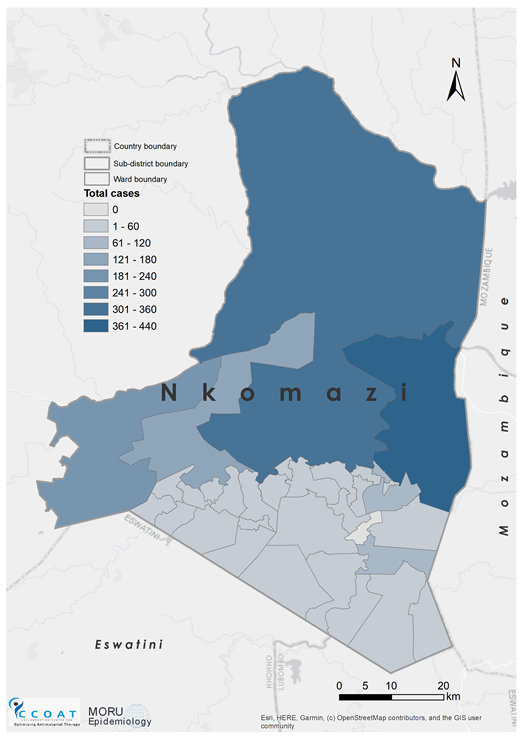a) | 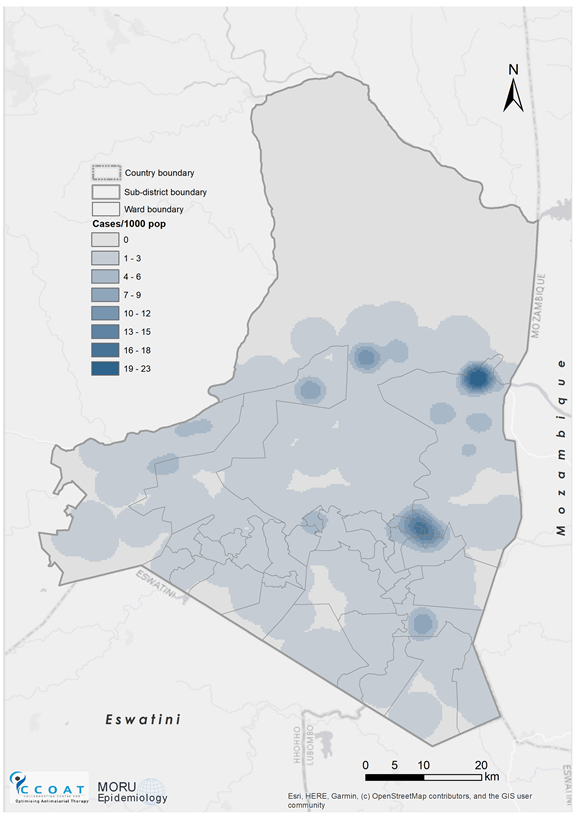b) |
| --- | --- |
| ***Figure S7:*** *Malaria case distribution maps evaluated for the study area; a) a thematic map of distribution of malaria cases by ward b) a 0.5 X 0.5 density map of cases. The latter was selected as suitable for further mapping malarial cases and drug resistance as a background shapefile.* | |

## **Tool S1. GPS tools for training malaria case investigators**

### Tool S1A: A training guide for trainers

| **Section 1: Introduction**  Strengthening surveillance activities towards accurate and comprehensive data and reporting is central in achieving malaria elimination. Apart from enhancing monitoring and planning activities, improving the quality of malaria surveillance immensely helps in prioritisation of resources. The 2016 – 2030 WHO strategy recommends surveillance to be adopted in the health system as a core intervention. Programmes need to focus resources in training their surveillance teams to be able to keep up with current requirements and the use of new technologies such as tablets and GPS machines. Coupled with the knowledge of the work flow of data through different health information systems such as DHIS2 (District Health Information Systems II) surveillance personnel with supervisory roles are gate-keepers and provide a major link between field data collection and programme management.  This training is therefore aiming at empowering surveillance case investigators in good and well-informed practice to perform case investigation using GPS devices.  This training should be carried out in three parts. A theoretical part should be completed in class first, with the aim of achieving the learning goals below and the second part should be a supervised field practical. The third part is for visualisation of the coordinates collected during the field practical, discussion and feedback sharing. This last part should also address the transcription and copying errors. |
| --- |
| **Tasks for the trainers/supervisors**  This training manual assumes the environment health officers and information officers to be fluent with case investigation, data flow and reporting, therefore be able to train the case investigators regularly to ensure high quality of data. |
| **Learning goals**   1. Each case investigator to understand the basic concepts in describing location and direction. 2. Each case investigator to understand the minimum requirements of case/household identifiers to be able to find a case’s household. 3. Each case investigator to be fully knowledgeable in understanding how to calibrate a GPS device, obtain coordinates and troubleshoot frequent location queries. 4. Each case investigator to be fluent in the information required, and be able to complete location information, in the case investigation form and the rationale of each input. 5. Each case investigator to be able to independently capture and copy down location correctly and understand how coordinates are visualised. |
| **Materials**  Functioning and powered GPS devices and/or tablets, scratch book, pens and papers.  Standard Operating Procedures for GPS and/or tablets.  Flip card or PowerPoint (PPT) presentation (attached) can be used to summarise the important information. |
| **Procedure**  **Part 1 – Basic concepts in telling and capturing location using GPS devices or tablets (15 – 20 mins)**  Identify the level of knowledge:  You can assess the knowledge gap by asking the team to respond to a prepared survey to understand how to customise your training. However, if that is time consuming you can use a consensus approach as below.  Begin by allowing case investigators to explain to you their role and field activities. Ask one or two to fully describe the procedure and ask if there any other alternatives to performing case investigation. Building on their view and addressing the gaps, use the PPT to guide you through the process.  Allow time for a discussion by asking few questions or responding to questions after every 2-3 slides.  Go through the PPT and discuss on the basics of describing location and direction. Use relevant examples to probe participants to describe and give directions of arriving at a place e.g. the training venue, nearby hospital or any famous structure or farm.  The next part will be going through the SOPs, spending between 20 – 30 mins for Garmin eTrex SOP reading, operating the device and discussion. Use the icons listed in each instruction to help guide the participants to arrive at the right setting. In groups of 2 -3 participants, allow 5 – 10 minutes for each group to individually go through the process again and discuss any challenges.  Use similar time for the chosen android application (e.g. *‘GPS Essentials’)* and follow the same procedure as above.  **Part 2A - Writing coordinates (10 mins discussion)**  By using the case investigation fields, discuss with case investigators on how the coordinates, direction and address information should be completed.  **Part 2B - Field data collection (30 mins exercise)**  Ask case investigators to go to the nearby well-known structures and record the addresses and coordinates on a paper.  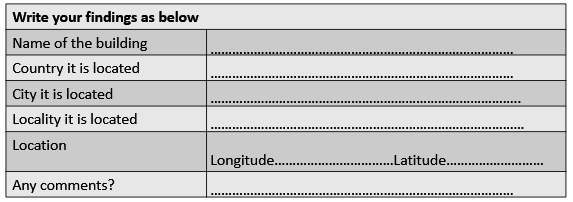  **Part 3 – Coordinates’ recording and visualisation (30 mins)**  Ask case investigators to swap their copied locations  Create an excel sheet and list the places as below.  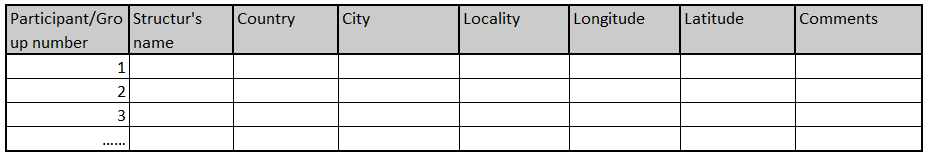  Use Google Maps to visualise these coordinates;   - Open Google Maps on your computer - Select ‘your locations’ - Select ‘maps’ - Select ‘create maps’ - Select ‘add layer’ - Select ‘upload file’ (here you will upload your excel sheet) and your coordinates will appear on the Google Maps - Zoom in to show the participants and probe if their locations have been captured correctly |
| **Assessment method**  You can send a post training survey (paper- or online based tool) to assess the previous identified knowledge gap and see if the learning goals have been achieved. You can also ask the participants the degree of satisfaction with the training and obtain their feedback for future use.  Alternatively, you can use the consensus approach for a general feedback to see if they have understood. Project the learning objectives slide and see how much they agree by show of hands to each learning goal. |
| **Other supportive information** |

### Tool S1B: Standard operating procedures for eTrex 10 GPS device (adapted from Health Geolab Collaborative, 2018) [1].

| \| 1. Plan your day:   Turn your GPS machine on and off to make sure there is enough battery for the day.   1. At your facility, identify the cases you will be investigating for the day and plan your site visits 2. When you arrive at the site for obtaining the GPS coordinates, first, identify and write down the name or address (stand/street number/ward/subdistrict), then, follow through the procedures below; \| \| --- \| \| 1. Find an **open space** and turn the GPS device on. \| \| 1. Click twice on the **menu** button on the left of the device to get to the menu page. \| \| 1. Scroll down the menu page using the thumbstick, until the "**Setup**" icon is highlighted. Next, press the thumbstick to access the Setup sub-menu. \| \| 1. Scroll down the menu page using the thumbstick, until the "System" icon is highlighted. Next, press the thumbstick to access the **System** sub-menu.    \| \| 1. Under "Satellite System" highlight the **"GPS+GLONASS"** option and press the thumbstick to save the selection.   *This will allow you to use both the GPS and GLONASS constellations of satellites as shown here*  ** \| | \| 1. Press the **back** button on the right of the device to return to the Setup sub-menu. \| \| --- \| \| 1. Use the thumbstick again to arrive at the "**Units**" icon is highlighted. Next, press the thumbstick to access the Units submenu \| \| 1. Set both the "**Distance and Speed**" as well as "**Elevation**" items to the metric system as shown here:    \| \| 1. Click on the **back** button on the right of the device to return to the Setup sub-menu. \| \| 1. Use the thumbstick again until the "**Position Format**" icon is highlighted. Next, press the thumbstick to access the Units sub-menu. \| \| 1.  Set the "**Position Format**," "**Map Datum**," and "**Map Spheroid**" as presented here: \| \| 1. Click on the **back** button twice to return to the menu page \| \| 1. Hold the GPS device horizontally in front of you, use the thumbstick to scroll down the menu page until the "**Satellite**" icon is highlighted: \| |
| --- | --- | --- | --- | --- | --- | --- | --- | --- | --- | --- | --- | --- | --- | --- | --- |

| \| 1. Next, press the thumbstick to access the "**Satellite page**" which looks as follows:   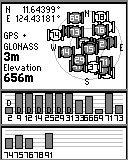 \| \| --- \| \| 1. Wait for the accuracy value to become less than **15 metres** with at least **4 satellite signals** received (number of grey bars at the bottom of the page). Stay for at least one minute at the same spot to allow for the best reading possible.   *In the example shown here, the accuracy is 10 m with 6 satellite signals received (the transparent bar is not counted).*   \| \| 1. If there is **no fixing** of the satellite signal, make sure you are under open sky and take a few (2 or 3) steps and repeat step 15 \| | \| 1. Once the accuracy value is below 15 metres with at least 4 satellite signals, **temporarily write down** the number of satellite signals and the accuracy measure on a piece of paper. \| \| --- \| \| 1. Mark the waypoint by pressing and holding the thumbstick. This will take you to the “**Mark Waypoint**” page as seen here:    \| \| 1. If this is not the case, this means that the coordinates fall outside the ranges.  **Recheck** the units and position format of the device (see steps 7 to 11 above). \| \| 1. **Note** the final number of satellite signals and accuracy to make sure there is a minimum of 4 satellites and the accuracy is within 15m. \| \| 1. Record the **coordinates** with 5 decimals in the case investigation form.   e.g. *Latitude (decimal degrees)  Longitude (decimal degrees)*   \| - \| 2 \| 5 \| . \| 4 \| 7 \| 9 \| 0 \| 8 \| \| --- \| --- \| --- \| --- \| --- \| --- \| --- \| --- \| --- \|  \| 0 \| 3 \| 0 \| . \| 9 \| 7 \| 2 \| 9 \| 4 \| \| --- \| --- \| --- \| --- \| --- \| --- \| --- \| --- \| --- \| \| \| 1. **Check**. Go back through the form and complete any missing fields. \| \| 1. Move to the next household and start again from step A. IMPORTANT: Turn off the GPS device by holding down the ‘**on/off’** button until you are at the next point to be collected. \| |
| --- | --- | --- | --- | --- | --- | --- | --- | --- | --- | --- | --- | --- | --- | --- | --- | --- | --- | --- | --- | --- | --- | --- | --- | --- | --- | --- | --- | --- | --- |

Tool S1C: Standard operating procedures for GPS-Essentials android application

| **Instructions for downloading, obtaining & recording coordinates using the ‘GPS Essentials’ App on an Android Mobile Phone or Tablet**  Shortened version:   \| 1. Download ‘GPS Essentials’ App from ‘Google Play Store’ 2. Customise the settings to; “Units” – meters (SI), “Position Datum” – World Geodetic System 1984, “Position format” – Decimal 3. Open “Satellites”, the App will display coordinates, satellites and accuracy. 4. Select “Dashboard” and add items you want to be displayed every time you are collecting coordinates. \| \| --- \|   Extended version:   \| 1. To download this App you will need; 2. An internet connection through WIFI or using data via your mobile subscriber. 3. A ‘Play Store account’, you can proceed with steps below if you already have an account. If you do not have it, open ‘Google Play Store’ and follow instructions on registering an account or proceed with the instructions below if you already have an account to download applications. \| \| \| --- \| --- \| \| 1. In the Play Store, click on the search window and type ‘GPS Essentials’ \| 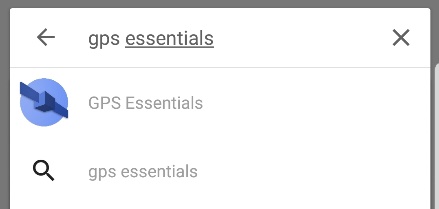 \| \| 1. Select the App and choose ‘INSTALL’ for it to be installed in your advice. \| \| \| 1. If you get a warning “Cannot download App from unknown sources”, go to settings>>allow downloads from unknown resources>>select to it to allow (on). \| \| \| 1. Wait for a few minutes, the App will download and install automatically. After the installation select ‘OPEN’ to have the app displayed in your tablet/phone. It will show the App menu as below. \| 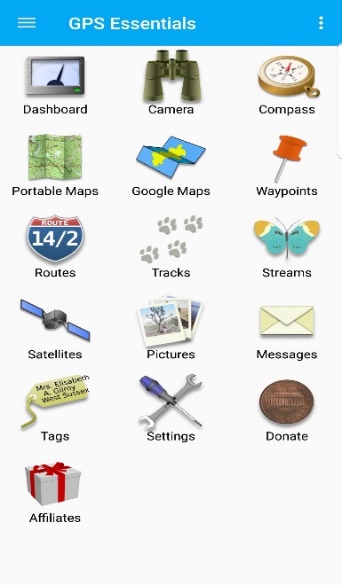 \| \| 1. Select “Satellites” and coordinates, precision and number of satellites will be displayed as below in two lines \| 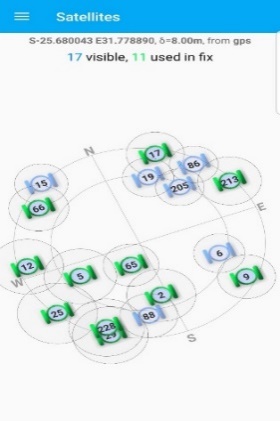 \| \| 1. Select the 3 lines on the left side corner to open the sliding submenu and scroll down to “Settings”. \| 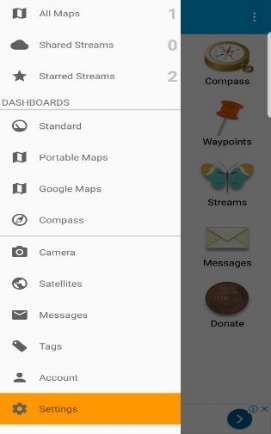 \| \| 1. In settings, scroll down to “**Presentation**” and select;    1. **Units** and set this to ***Meters (SI)***    2. **Position Datum** and set it to ***World Geodetic System 1984***    3. **Position format** and set it to ***Decimal*** \| \| \| 1. Click the back arrow to get back to the main Menu and select **“Dashboard”**. Here we will add a dashboard to display our readings as follows: select the plus **‘+’** item on the right upper side of the screen to open the dashboard submenu with the items to select.    1. ***Accuracy***    2. ***Date***    3. ***Latitude***    4. ***Longitude***    5. ***Number of Satellites*** \| \| \| *Note on the left side the colour highlights on the left side when an item gets selected.* Then choose ‘close’ to end the submenu window \| \| \| 1. Your dashboard will be ready with the five items to display always when collecting coordinates. \| 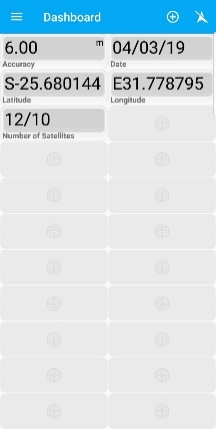 \| \| When you arrive at the point for collecting coordinates. Open the ‘GPS Essentials’ and select dashboard to get the reading. Record the coordinates on your paper forms. \| \| |
| --- | --- | --- | --- | --- | --- | --- | --- | --- | --- | --- | --- | --- | --- | --- | --- | --- | --- | --- | --- | --- | --- | --- | --- | --- | --- |

## **Tool S2. Treatment adherence and response form**


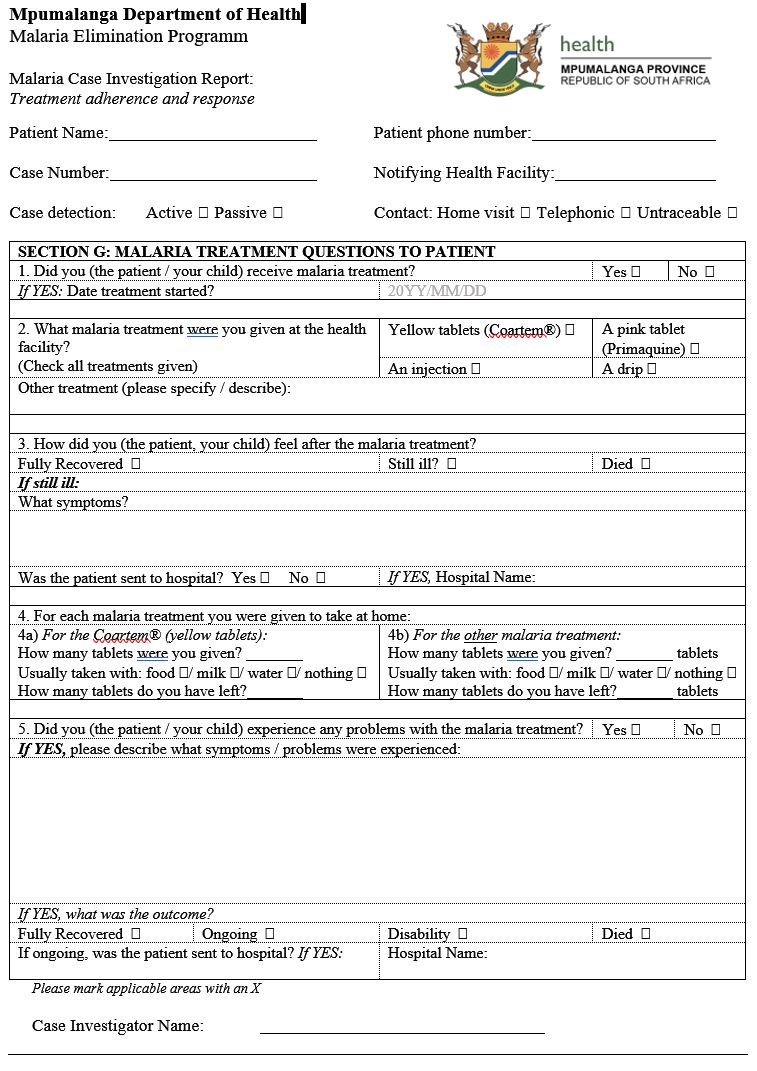


References

1. Ebener S, Maude RJ, Gault P. Guidance for the management and use of geospatial data and technologies in health. Health Geolab Collaborative; 2018.
